# Visualizing Collaboration Characteristics and Topic Burst on International Mobile Health Research: Bibliometric Analysis

**DOI:** 10.2196/mhealth.9581

**Published:** 2018-06-05

**Authors:** Lining Shen, Bing Xiong, Wei Li, Fuqiang Lan, Richard Evans, Wei Zhang

**Affiliations:** ^1^ School of Medicine and Health Management, Tongji Medical College Huazhong University of Science & Technology Wuhan China; ^2^ Institute of Smart Health Huazhong University of Science & Technology Wuhan China; ^3^ Hubei Provincial Research Center for Health Technology Assessment Wuhan China; ^4^ Department of Business Information Management and Operations University of Westminster London United Kingdom

**Keywords:** collaboration characteristics, topic bursts, international mobile health, mHealth, telemedicine, bibliometric analysis, bibliometrics, research trends

## Abstract

**Background:**

In the last few decades, mobile technologies have been widely adopted in the field of health care services to improve the accessibility to and the quality of health services received. Mobile health (mHealth) has emerged as a field of research with increasing attention being paid to it by scientific researchers and a rapid increase in related literature being reported.

**Objective:**

The purpose of this study was to analyze the current state of research, including publication outputs, in the field of mHealth to uncover in-depth collaboration characteristics and topic burst of international mHealth research.

**Methods:**

The authors collected literature that has been published in the last 20 years and indexed by Thomson Reuters Web of Science Core Collection (WoSCC). Various statistical techniques and bibliometric measures were employed, including publication growth analysis; journal distribution; and collaboration network analysis at the author, institution, and country collaboration level. The temporal visualization map of burst terms was drawn, and the co-occurrence matrix of these burst terms was analyzed by hierarchical cluster analysis and social network analysis.

**Results:**

A total of 2704 bibliographic records on mHealth were collected. The earliest paper centered on mHealth was published in 1997, with the number of papers rising continuously since then. A total of 21.28% (2318/10,895) of authors publishing mHealth research were first author, whereas only 1.29% (141/10,895) of authors had published one paper. The total degree of author collaboration was 4.42 (11,958/2704) and there are 266 core authors who have collectively published 53.07% (1435/2704) of the total number of publications, which means that the core group of authors has fundamentally been formed based on the Law of Price. The University of Michigan published the highest number of mHealth-related publications, but less collaboration among institutions exits. The United States is the most productive country in the field and plays a leading role in collaborative research on mHealth. There are 5543 different identified keywords in the cleaned records. The temporal bar graph clearly presents overall topic evolutionary process over time. There are 12 important research directions identified, which are in the imbalanced development. Moreover, the density of the network was 0.007, a relatively low level. These 12 topics can be categorized into 4 areas: (1) patient engagement and patient intervention, (2) health monitoring and self-care, (3) mobile device and mobile computing, and (4) security and privacy.

**Conclusions:**

The collaboration of core authors on mHealth research is not tight and stable. Furthermore, collaboration between institutions mainly occurs in the United States, although country collaboration is seen as relatively scarce. The focus of research topics on mHealth is decentralized. Our study might provide a potential guide for future research in mHealth.

## Introduction

### Background

With continued economic and societal development worldwide, the traditional system of health care delivery has increasingly failed to satisfy human demand in providing efficient health care services. It should be noted that numerous constraints and barriers exist to providing high-quality, accessible, and timely health services, especially in low-resource settings [1-3]. In this context, mobile technologies have been introduced into health care service delivery, and, subsequently, mobile health (mHealth) has emerged, changing the situation by offering support via mobile communication technologies [4].

mHealth is an umbrella term that encompasses areas of networking, mobile computing, medical sensors, and other communication technologies within health care [5]. The first occurrence of the term “mHealth” in literature was in the special issue entitled “Unwired e-med” on wireless telemedicine systems, published in 2000 [6]. The World Health Organization Global Observatory for eHealth defines mHealth as “Medical and public health practice supported by mobile devices, such as mobile phones, patient monitoring devices, personal digital assistants (PDAs), and other wireless devices. mHealth involves the use and capitalization on a mobile phone’s core utility of voice and short messaging service (SMS) as well as more complex functionalities” [7]. Obviously, mHealth technologies can facilitate more accessible and affordable health care to all; it has presented unprecedented advantages over the past years [8]. Subsequently, it has attracted great attention from scholars, experiencing rapid development in recent years, and has become a hot topic in the health care field.

Given the importance of mHealth, some scientific researchers have focused on reviewing related literature to identify the characteristics and status of mHealth research in recent years. However, much of this effort has only considered specific subfields of mHealth, with conclusions being drawn from descriptive analysis and systematic reviews. For example, some reviews have focused on mobile health apps [9,10] related to the most prevalent conditions (eg, headache disorders [11], heart failure [12], HIV/AIDS [13]), and short message service (SMS) text messaging for health improvement [14-16]. Other reviews have concentrated on the analysis of mobile health technologies [17] and mobile devices for assessment of physical activity [18]. In addition, some scholars have summarized lessons learnt from mHealth trials and studies using peer-reviewed journals, websites, and key reports [19]. However, a review of previous related literature shows some research limitations. There have been few papers that have focused on the bibliometric perspective of mHealth research, which refers to methods of analyzing the data of scientific literature quantitatively, to gain knowledge of the meta-information related to the research in question [20,21]; the combined use of methodologies that give information on different aspects of scientific output is generally recommended [22]. In addition, discussion relating to the collaborative status and overall topic burst still remains relatively scarce.

### Objectives

The aim of this study, therefore, was to address these limitations by conducting a comprehensive exploration and analysis into the worldwide mHealth field, using quantitative analysis. Through this approach, major problems can be identified and raised. That is, what are the external characteristics of mHealth research, such as the growth in published literature and journal distribution? What is the status of collaboration between scholars in the field and trends in international mHealth research at the author, institution, and country level? What is the evolutionary process of the term bursts based on the high-frequency and highly bursting keywords set? What are the research topic bursts? The answers to these questions will not only supplement the previous research work completed but also contribute to further research on international mHealth.

## Methods

### Data Collection

In this study, we identified publications that are indexed in the Thomson Reuters Web of Science Core Collection (WoSCC) database, namely, the Science Citation Index Expanded, the Social Sciences Citation Index, and the Emerging Sources Citation Index. As WoSCC comprised most high-quality literature, and being updated continuously and dynamically, it has been identified as being most appropriate for the bibliometric analysis in this study [23].

To retrieve mHealth-related publications, as fully as possible, we formulated the following search strategy, on the basis of the above definition and reviews on mHealth (for further details on the search strategy employed, see Multimedia Appendix 1): #1 mobile health, #2 mHealth apps, #3 TS=((“mobile technolog*” OR “mobile device*”) AND “health*”), #4 TI=((“mobile phone*” OR “tablet comput*” OR “personal digital assistant*”) AND “health*”), #5 TI=(“mobile unit*” OR TI=“mobile health unit*”), #6 (#1 OR #2 OR #3 OR #4) NOT #5. Moreover, “document type” was limited to paper. The time span of publication was confined from 1985 to 2016.

**Figure 1:**
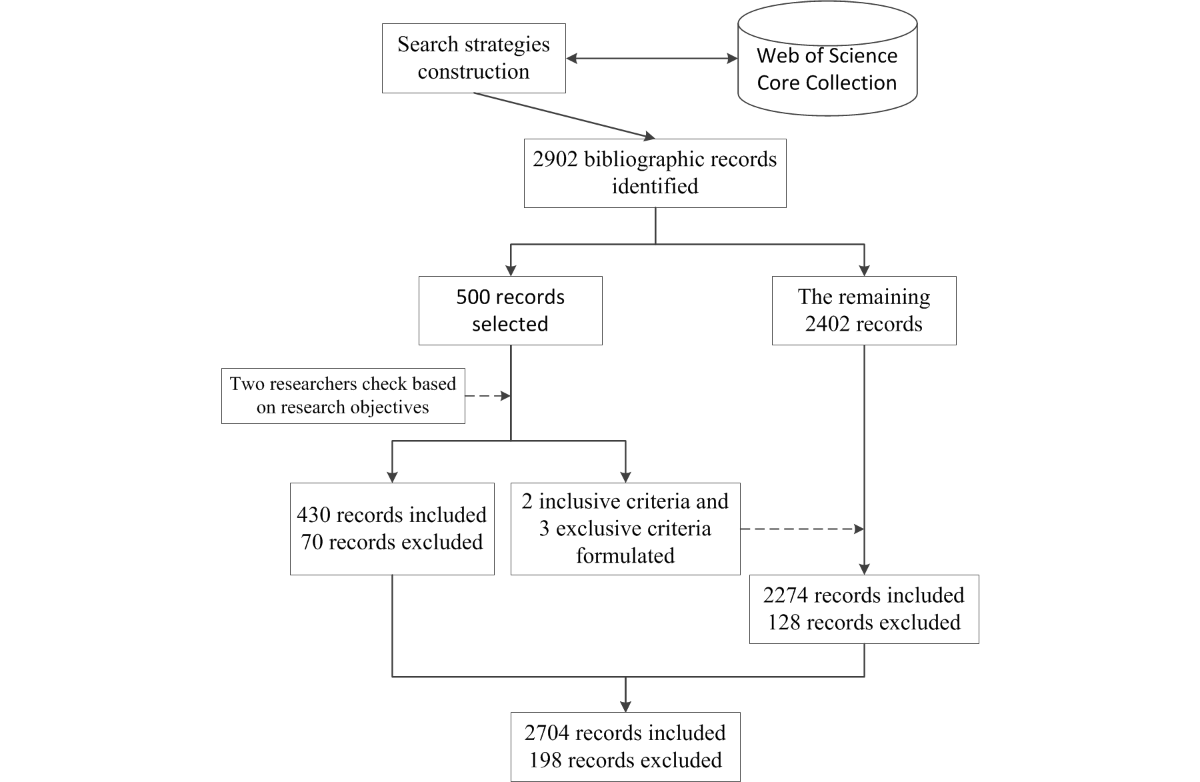
Selection process for obtaining bibliographic records on mHealth research.

On the basis of the above search strategy and restrictions, a total of 2902 bibliographic records were identified and downloaded on December 28, 2016. To perfect the research, the main inclusion and exclusion criteria were formulated after 2 researchers independently reviewed and evaluated the 500 pilot bibliographic records. The inclusion criteria were as follows: (1) the contents of the papers primarily concentrated on mobile health, and (2) all study designs. The exclusion criteria were as follows: (1) the record related to book review and notifications, instead of being a regular paper; (2) the content of the research focused on animal mobile health (eg, cattle [24]), rather than being focused on human-oriented mobile health; and (3) the study mainly concentrated on mobile units (eg, mobile health facilities [25]), rather than integration of Information and Communication Technologies (ICT) with health care services. In this process, the titles, abstracts, and keywords of the publications in these records were screened with reference to the research objective. Any discrepancies or disagreements were discussed until consensus was reached. Then, 1 researcher screened the remaining records using the above selection criteria. Finally, a total of 198 irrelevant records were manually removed. In total, 2704 bibliographic records published from 1997 through 2016 were obtained for subsequent bibliometric analysis, so as to cast light on collaboration characteristics and research topic burst in the field of international mHealth. The entire selection process of bibliographic records on mHealth research is shown in Figure 1.

### Design of Data Analysis Method

Similar to other bibliometric studies [26], a variety of analytic indicators have been employed in this research. Generally, bibliometric analysis can be used to depict and predict research trends and the direction of a given topic in a given field [27]. In this study, we analyzed literature distribution, including the growth in mHealth literature and journal distribution, using Bibliographic Item Co-occurrence Mining System (BICOMS) [28] and MS Excel 2010. In addition, core journals were identified, which normally refers to the most important journals with higher citation counts. That is, these core journals publish papers more frequently at a high academic level, which reflect the latest research findings, frontier research status, and developing trends of the subject; they are typically paid more attention by scientific researchers in the same research field.

In this research, the total number of published papers is regarded as an index of quantity of research productivity, whereas citation frequency is considered as an index of quality of research productivity. Therefore, the total local citation score (TLCS) and the total global citation score (TGCS) were calculated in this study. TLCS refers to the number of times that a set of papers included in a collection has been cited by other papers within the same collection, whereas TGCS refers to the number of times that a set of papers included in a collection has been cited in the WoSCC [29]. The average global citation score (AGCS) is the mean value of TGCS, which also indicates the average number of citations that papers in the mHealth field receive. Similarly, the average local citation score (ALCS) is the mean value of TLCS, which indicates the average number of citations that papers within the collection receive. In general, TLCS and TGCS have been the key indicators capable of evaluating the relevance of each research paper in our sample [30]. It is obvious that TLCS and TGCS can help us identify the most significant work on the topic. However, it should be noted that TLCS presents the important papers for a chosen research area, whereas TGCS mainly displays the effects of the papers related to a chosen research area on the papers in the WoSCC.

On the basis of the above indicators, HistCite, an analytical and visualization tool [31], was employed to analyze the research productivity of authors, institutions, and countries. Generally speaking, country collaboration, institution collaboration, and author collaboration are 3 primary forms of scientific collaboration. Coauthorship is fundamental in country collaboration and institution collaboration [32].

The rate of collaborative papers published is defined as the proportion of collaborated papers to the total number of papers, whereas the degree of author collaboration refers to the average number of authors per paper during a certain period of time; both indicators reflect the trend in collaborative research, to some extent [33].

CiteSpace Ⅱ [34] was used to directly visualize the 3 collaboration relationships. Visual maps generated by CiteSpace are composed of nodes and links. The node displays in a purple circle; nodes normally represent the author, institution, country, and so on, whereas links represent cocitation or co-occurrence between these nodes. On the basis of Chen’s definition [35], the higher citation and centrality the node has, the larger impact the node has in the cocitation map. By studying these clusters and the relationships between them, valuable information can be drawn. Finally, 4 stages were completed, as follows, regarding the analysis of research hotspots.

First, we calculated the frequencies of each keyword and created a coword matrix using BICOMS. When we considered equivalent relations between keywords, a total of 5543 keywords were identified from the publications and subsequently merged to obtain more precise results based on the following 3 principles: (1) merging of some keywords, which are entry terms, into corresponding Medical Subject Headings terms using PubMed (eg, “mobile phone,” “cellular phones,” and “cellular telephone” were merged into “cell phones”); (2) replacement of the full keyword into its acronym (eg, “Personal Digital Assistant” was replaced with “PDA”); and (3) merging of singular and plural keywords (eg, “mobile technology” and “mobile communication” were changed to “mobile technologies” and “mobile communications,” respectively). Then, 139 keywords, with the frequency not less than 10, were chosen to generate a 139 × 139 co-occurrence matrix. It should be noted that the data in diagonal cells were treated as missing data, and the values of nondiagonal cells were the co-occurrence frequency [36].

Second, burst detection was conducted on the cleaned bibliographic records, and a temporal bar graph for keywords was drawn. Kleinberg’s burst detection algorithm [37], which can identify sudden increases or “bursts” in the frequency of words used over time, is effective in detecting bursts in keyword popularity. We employed Science of Science (Sci2) [38], which can implement such algorithm, to detect the burst terms in the cleaned bibliographic records and calculate the burst strength which depicts the intensity of the burst, that is, how great the change is in the word frequency that triggered the burst. In total, 228 keywords with a burst strength not less than 1 were generated. However, these keywords only represented the possibility to be core keywords and needed to be further selected, according to the keyword frequency that reflects the degree of concern to some extent. The higher the number of keyword frequency, the more likely it is to become a hot topic in future. We further computed the intersection of the high-frequency (frequency≥10) keywords set and highly bursting (burst strength≥1) keywords set [39], so as to reduce the interference caused by low frequency keywords. As a result, 71 keywords were obtained. Next, a temporal visualization map for the 27 keywords with a frequency not less than 10 and burst strength not less than 2 was drawn using Sci2. Each row record is represented as a horizontal bar with a specific start and end date, with a corresponding keyword label on its left side in the temporal bar graph visualization. The area of each bar encodes a numerical value of burst strength.

Third, hierarchical cluster analysis was conducted, based on the 71 × 71 co-occurrence matrix. At first, we removed any rows or columns that did not correspond to any 1 of the 71 keywords from the 139 × 139 co-occurrence matrix. Finally, the 71×71 co-occurrence matrix was formed and then transformed into Pearson’s correlation matrix, using IBM SPSS Statistics 19. In this matrix, every value in the cell indicates the similarity of each keyword pair [40]. Considering the discrete matrix data, a dissimilarity matrix was created. Subsequently, hierarchical cluster analysis was performed using SPSS 19.0 [41], and the results display directly the keywords cluster.

Finally, the visualization map and its network characters were obtained by analyzing the original Pearson’s correlation matrix, using MS Excel 2010 and Ucinet6.6 [42,43]. The density of the network was calculated, with a social network map being drawn, using Ucinet6.6 and Netdraw (embodied in the Ucinet tool), to verify the result above. Furthermore, the relative size of nodes is proportional to the frequency of keywords, whereas the relative thickness of lines is drawn proportionally to the correlation between keywords [44].

## Results

### Literature Distribution

#### Growth of Literature

**Figure 2:**
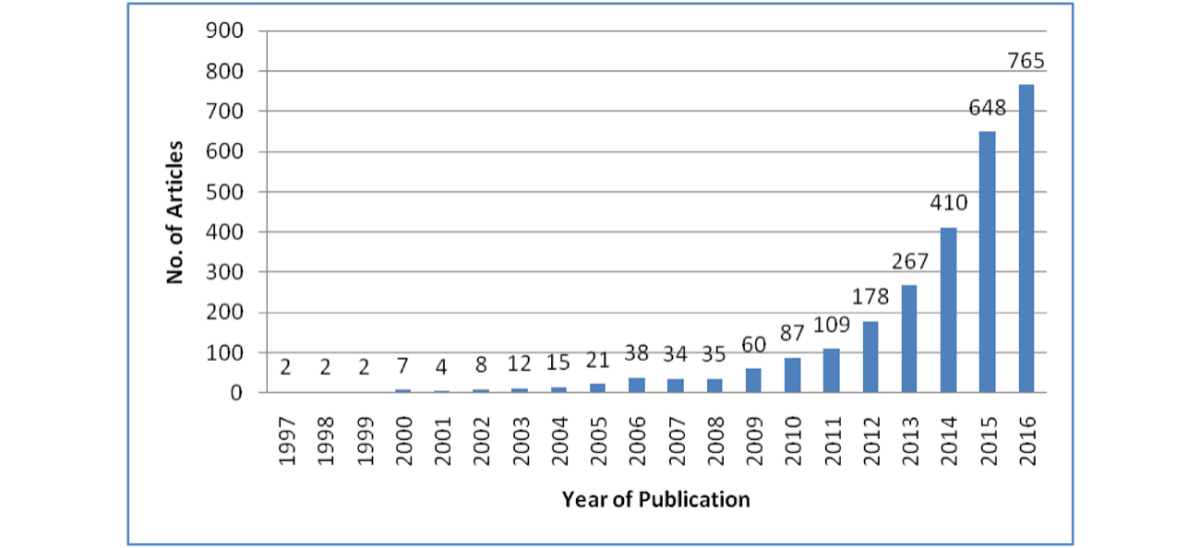
Number of publications related to mHealth in Web of Science Core Collection (1997-2016).

On the basis of the above search strategy and the cleaned data obtained, we found that the earliest paper on mHealth, indexed by WoSCC, was published in 1997. The publication output of mHealth-related research, from 1997 to 2016, is presented in Figure 2, indicating that the number of papers concerning mHealth research has risen yearly and produced from 2 in 1997 to 765 in 2016. In terms of publication language, most (98.08%, 2652/2704) are written in English, followed by German, Portuguese, and Spanish. Since the beginning of 2012, it should be recorded that the number of mHealth-related publications has increased considerably.

**Figure 3:**
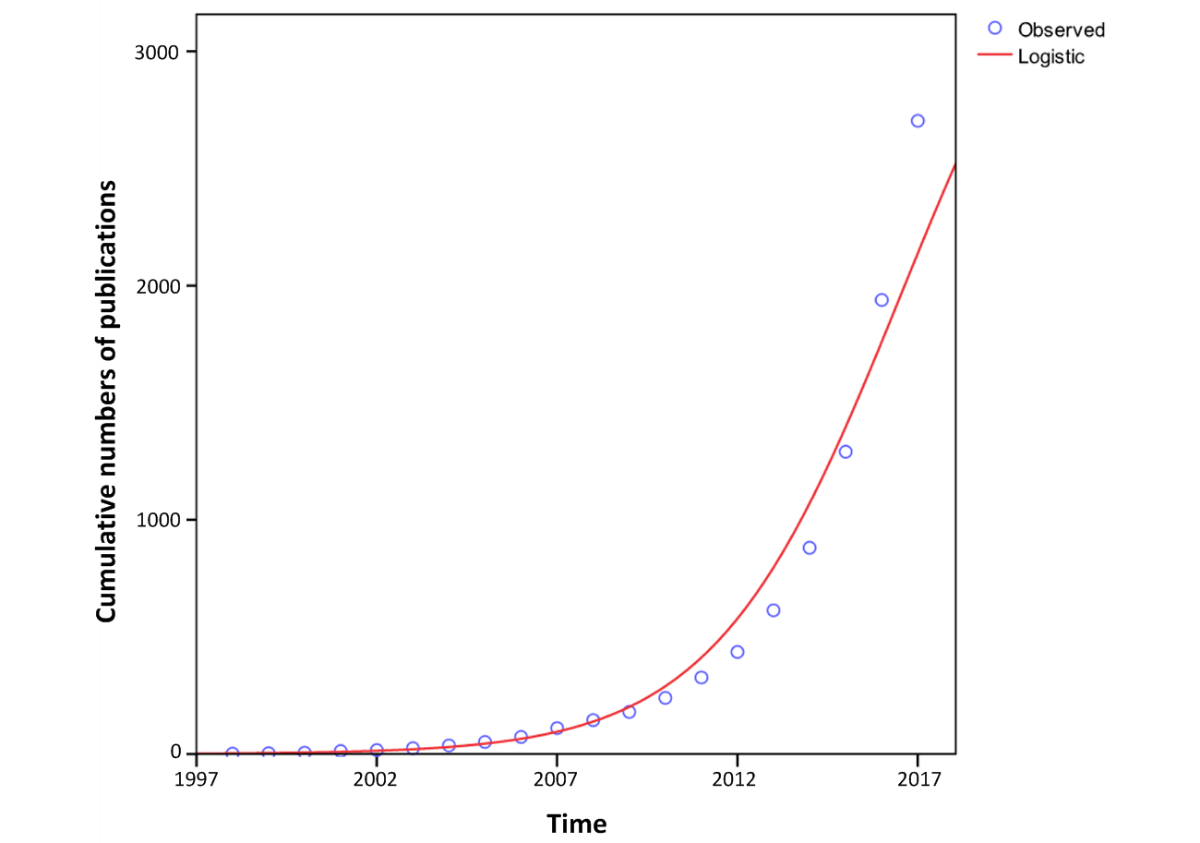
The relationship between cumulative number of publications and years since 1997.

The cumulative annual number of publications has continually grown from 2 to 2704 (shown in Figure 3). A literature logical growth curve was obtained by a direct fit to the equation: *y* = 3913.14 / (1 + 1929.18e^-0.39t^), (*R*^2^>0.987), where *y* is the cumulative annual number of papers and *t* is the number of years since 1997. The time of the inflection point of the growth curve is: *t* = *ln* (1929.18) / 0.39 = 19.4 ≈ 20 (ie, 2016 - 1997 + 1).

#### Journal Distribution

From 1997 to 2016, research relating to mHealth has been published in 1008 journals. These journals were listed in a descending order by the productivity of publication and then divided into a nucleus of journals and 2 following groups, containing approximately the same number of publications as the nucleus. Note, the “Journal of Medical Internet Research” is the most productive journal, publishing a total of 125 papers on mHealth research.

As shown in Table 1, the nucleus, covering the Top 18 journals (1.79%, 18/1008), has 853 papers, accounting for 31.55% of all 2704 papers. The relationship among the number of journals in the nucleus and the 2 succeeding zones is approximately 1:7:7^2^; this follows Bradford’s Law of scattering [45].

**Table 1:** Top 18 journals (by article count) on the topic of mobile health (mHealth).

No.	Top journals	IF^a^ (2015)	IF (2016)	Articles, n (%)	Cumulative percentage
1	*Journal of Medical Internet Research*	4.532	5.175	125 (4.62)	4.62
2	*Telemedicine and E-Health*	1.791	2.031	120 (4.44)	9.06
3	*JMIR mHealth and uHealth*	N/A^a^	4.636	107 (3.96)	13.02
4	*Journal of Medical Systems*	2.213	2.456	71 (2.63)	15.64
5	*PLoS ONE*	3.057	2.806	49 (1.81)	17.46
6	*BMC Medical Informatics and Decision Making*	2.042	1.643	38 (1.41)	18.86
7	*International Journal of Medical Informatics*	2.363	3.210	38 (1.41)	20.27
8	*Journal of the American Medical Informatics Association*	3.428	3.698	37 (1.37)	21.63
9	*BMC Public Health*	2.209	2.265	36 (1.33)	22.97
10	*Journal of Telemedicine and Telecare*	1.377	2.008	34 (1.26)	24.22
11	*JMIR Research Protocols*	N/A^b^	N/A	32 (1.18)	25.41
12	*IEEE Transactions on Information Technology in Biomedicine*	N/A	N/A	32 (1.18)	26.59
13	*IEEE Journal of Biomedical and Health Informatics*	2.093	3.451	28(1.04)	27.63
14	*Journal of Health Communication*	2.013	1.614	24 (0.89)	28.51
15	*Trials*	1.859	1.969	24 (0.89)	29.40
16	*Health Informatics Journal*	1.578	3.021	21 (0.78)	30.18
17	*Sensors*	2.033	2.677	19 (0.70)	30.88
18	*Personal and Ubiquitous Computing*	1.498	2.395	18 (0.67)	31.55

^a^IF: impact factor.

^b^N/A: not applicable.

The journal impact factor (IF), in a given year, is defined as the number of citations received by papers published in the previous 2 years, divided by the number of papers published in the same time. Table 1 shows that the journal IF rose in 2016 for 13 of the 18 top journals, when compared with 2015, except for 4 journals, namely, *PLoS ONE, BMC Medical Informatics and Decision Making, Journal of Health Communication, IEEE Transactions on Information Technology in Biomedicine, and JMIR Research Protocols.* Moreover, the average IF of the top 16 journals in 2016 reached 2.82. A total of 9 of the 18 journals are in the category of Medical Informatics in the Journal Citation Reports 2016.

### Collaboration Characteristics

#### Core Author and Author Collaboration

The total number of authors who have published research in the field of mHealth is 10,895, 21.27% (2318/10,895) of which have been as first author. However, 141 authors have published just 1 paper, comprising 1.29% (141/10,895) of the total. The top 7 most productive first authors with not less than 5 outputs were identified in the area of mHealth (shown in Table 2), which together contributed to the publication of 45 papers (for list of papers published, see Multimedia Appendix 2), that is, an average of 6.4 papers per first author during the period of 1997-2016. Table 2 also shows that the most productive first author in the field of mHealth is John D Piette with 11 papers, followed by Dror Ben-Zeev and David D Luxton.

In this study, a total of 2563 coauthored papers were identified, indicating that the rate of collaborative papers is 94.79% (ie, 2563/2704). The total publication frequency of authors, which refers to the cumulative result of the number of authors of each paper, is 11,958, indicating that the degree of author collaboration was 4.42 (11,958/2704) during the period of 1997 to 2016.

**Figure 4:**
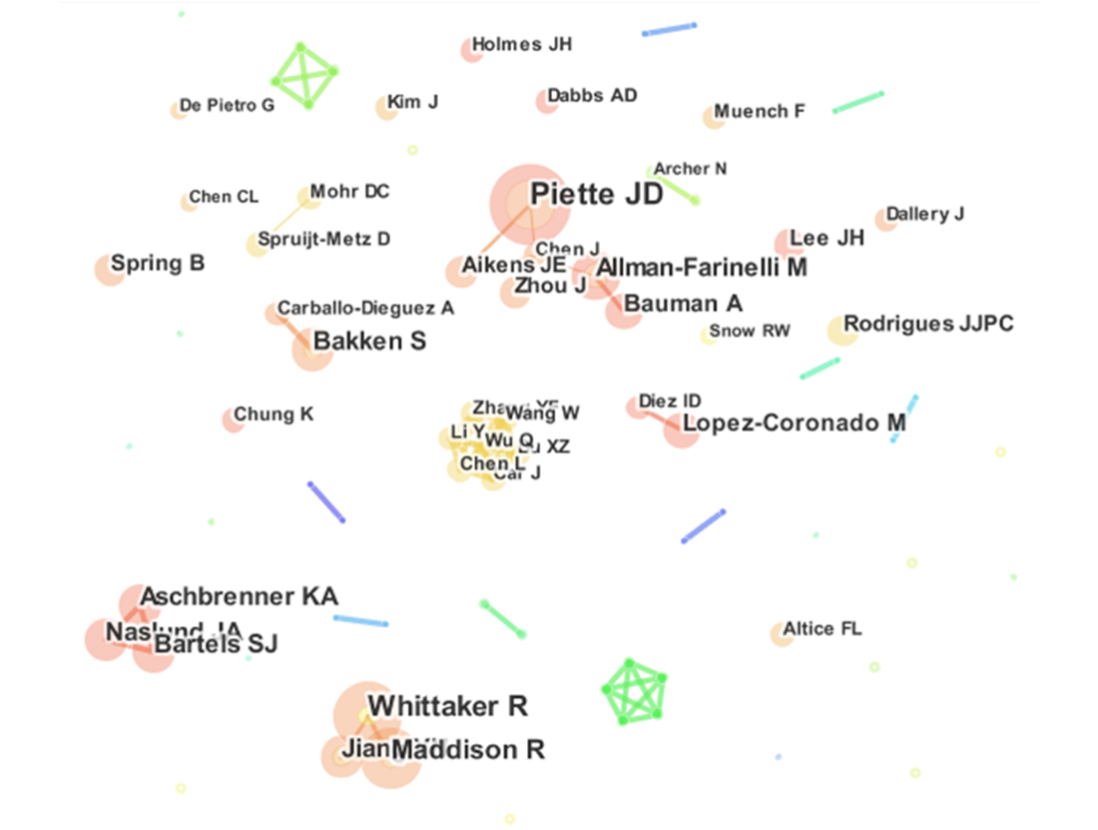
The collaboration relationship of productive authors publishing mHealth research.

The visualization network of author collaboration was created using CiteSpace based on the g-index selection criteria in each slice (shown in Figure 4). Note that several authors tended to collaborate with a small group of collaborators, generating 4 major clusters with some highly active authors. Namely, Cluster 1, takes the top spot, which includes 5 core members, including Piette JD, Allman-Farinelli M, Bauman A, Aikens JE, and Chen J; Cluster 2 consists of Whittaker R, Maddison R, and Jiang YN; Cluster 3 contains Aschbrenner KA, Naslund JA, and Bartels SJ; and the core members of cluster 4 are Wang W, Wu Q, Chen L, and Li Y. Additionally, there are a large number of relatively smaller clusters in the collaborative map of authors (for list of relevant information for main authors of the 4 clusters, see Multimedia Appendix 3).

**Table 2:** The top 7 most productive first authors during the period 1997-2016.

Author name (full name)	ORCID^a^	Recs-first^b^ (Recs-all^c^)	Percentage^d^	Main affiliation	Country
Piette JD (John D Piette)	N/A^e^	11 (20)	0.41	Ann Arbor Department of VA, Center for Clinical Management Research, Michigan	United States
Ben-Zeev D (Dror Ben-Zeev)	0000-0001-6597-2407	8 (10)	0.30	Dartmouth Medical School, Hanover	United States
Luxton DD (David D Luxton)	N/A	6 (6)	0.22	The National Center for Telehealth and Technology, Tacoma, Washington	United States
Chib A (Arul Chib)	N/A	5 (5)	0.18	Nanyang Technological University	Singapore
Turner-McGrievy, GM (Gabrielle M Turner-McGrievy)	0000-0002-1683-5729	5 (7)	0.18	University of South Carolina, Columbia, South Carolina	United States
Aschbrenner KA (Kelly A Aschbrenner)	N/A	5 (9)	0.18	Geisel School of Medicine at Dartmouth, Lebanon, NH	United States
Akter S (Shahriar Akter)	0000-0002-2050-9985	5 (5)	0.18	University of Wollongong	Australia

^a^ORCID: Open Researcher and Contributor ID.

^b^Recs-first: number of papers published as first author.

^c^Recs-all: total number of papers published by the author.

^d^Percentage: Percentage of papers published as first author.

^e^N/A: not applicable.

The core authors group is recognized as those authors with more publications and influence than others. On the basis of Price Law [46] (for the equation, see Multimedia Appendix 4), the minimum output of core author is obtained, namely, approximately 3.35 (ie, 0.749 x 20^1/2^), which means that the publication output of every core author is not less than 4. From this research, we can identify 266 core authors who have collectively published 1435 papers or 53.07% (1435/2704) of the total number of publications.

#### Institution and Collaboration

Statistical data analysis shows that the 2704 identified publications in the mHealth field were distributed among 3040 institutions. As shown in Table 3, authors from top 10 research institutions have published 449 (16.61%, 449/2704) papers. The University of Michigan performed well, being seen as the most productive institution in mHealth research, followed by The University of Washington and Harvard University. All 10 institutions are universities, with 9 being based in the United States. The TLCS and TGCS of the University of Washington can be seen as the highest among the universities. Harvard University has the highest AGCS, with high academic influence and collaboration in mHealth research, followed by the University of Washington.

**Figure 5:**
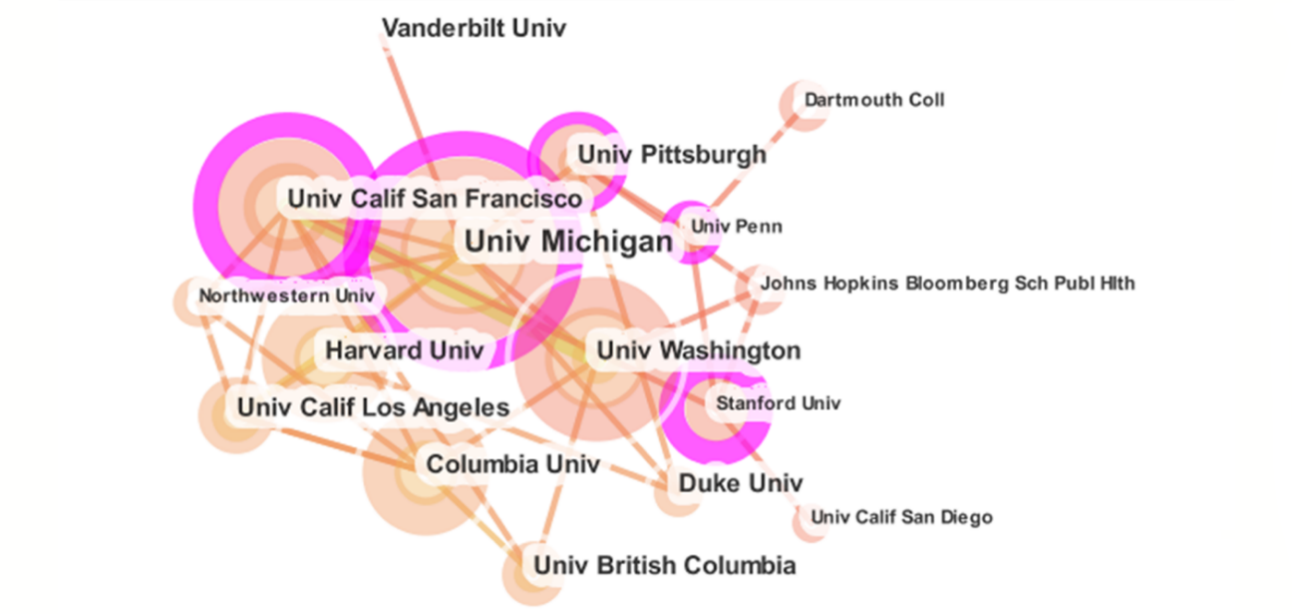
The collaboration relationship between institutions related to mHealth research.

Compared with other forms of collaboration, institutional collaboration provides a measure to examine the interactions between institutions on a more granular level [47]. After being pruned [48], the major collaboration relationship of institutions related to mHealth research is shown in Figure 5, in which the institution labeling is shown based on the citation frequencies with 20 threshold levels (for list of the corresponding relations between the abbreviations and the full forms of the main institutions, see Multimedia Appendix 5). It is noted that there are 5 universities that present higher centrality with the purple circle, namely: The University of Michigan, University of California San Francisco, Stanford University, University of Pittsburgh, and The University of Pennsylvania, which demonstrated the central position and academic importance in the collaborative network of mHealth research. The links between institutions are relatively few, which coincides with the foregoing analysis.

#### Country and Collaboration

In total, scholars from 111 countries and territories have contributed to research on mHealth. A total of 10 countries and territories have contributed to the publication of 2477 papers (shown in Table 4). The United States, which is the most productive country in mHealth research, ranks the first in publication outputs, accounting for 46.97% (1270/2704) of the total. The United Kingdom, Australia, Canada, and China are not far behind. Moreover, when combining with the report released by the World Bank [49], it can be acknowledged that there are 20 lower middle-income countries (LMICs) that have contributed to mHealth research. A total of 197 (7.29%, 197/2704) papers were contributed to by authors in LMICs.

Furthermore, the TLCS and TGCS of the United States are the highest, followed by the United Kingdom and Canada. The top 6 countries in a descending order by AGCS, which indicates the high average quality of these papers, are the United States, Canada, the United Kingdom, China, Australia, and Germany.

**Figure 6:**
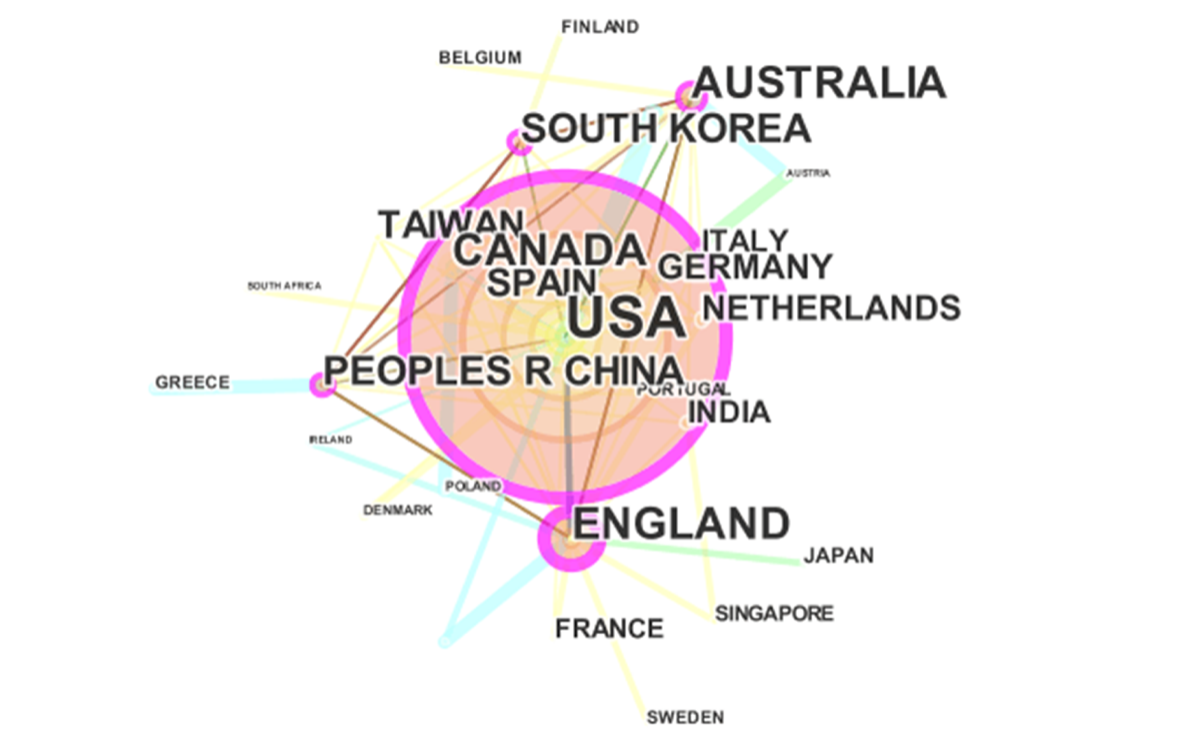
The collaboration relationship of country and territory related to mHealth research.

Figure 6 shows the collaboration relationship of the most productive countries and territories. Country and territory labeling is shown based on the citation frequencies with 10 threshold levels (for list of the corresponding relations between the abbreviations and the full forms of the main countries and territories, see Multimedia Appendix 6). The United States is obviously the most active country in mHealth research worldwide. In the mHealth field, the United States plays an irreplaceable leading role, although the collaboration of authors inside the country is relatively scarce. It is also noteworthy that there are another 4 countries and territories that demonstrate higher centrality with the purple circle, namely, England, Australia, South Korea, and China.

**Table 3:** Top 10 institutions on mobile health (mHealth) research.

No.	Institution	Recs^a^	Publication, %	Cumulative percentage	TLCS^b^	TGCS^c^	AGCS^d^
1	University of Michigan	60	2.22	2.22	101	462	7.70
2	University of Washington	56	2.07	4.29	176	818	14.61
3	Harvard University	53	1.96	6.25	91	775	14.62
4	University of California, San Francisco	48	1.78	8.03	56	401	8.35
5	Columbia University	44	1.63	9.65	66	275	6.25
6	University of Sydney	40	1.48	11.13	42	339	8.48
7	Johns Hopkins Bloomberg School of Public Health	39	1.44	12.57	46	220	5.64
8	University of California, Los Angeles	39	1.44	14.02	83	540	13.85
9	Johns Hopkins University	35	1.29	15.31	22	137	3.91
10	University of Pittsburgh	35	1.29	16.60	84	334	9.54

^a^Recs: number of published papers.

^b^TLCS: total local citation score.

^c^TGCS: the total global citation score.

^d^AGCS: average global citation score.

**Table 4:** Top 10 countries and territories.

Country and territory	Recs^a^	TLCS^b^	TGCS^c^	ALCS^d^	AGCS^e^
United States	1254	1721	11648	1.37	9.30
United Kingdom	263	171	2141	0.65	8.14
Australia	178	150	1371	0.84	7.70
Canada	175	243	1583	1.39	9.05
People’s Republic of China	136	98	1061	0.72	7.80
South Korea	116	62	577	0.53	4.97
Spain	106	39	573	0.37	5.41
Taiwan	88	94	818	1.07	9.30
Germany	83	27	499	0.33	6.01
Netherlands	78	36	442	0.46	5.67

^a^Recs: number of published papers.

^b^TLCS: total local citation score.

^c^TGCS: the total global citation score.

^d^ALCS: average local citation score.

^e^AGCS: average global citation score.

### Research Hotspots

#### Temporal Bar Graph for High-Frequency and High-Burst Keywords

There are 71 keywords with a burst strength more than 1 and frequency not less than 10, which were ranked according to the frequency of keyword (for the details, see Multimedia Appendix 7). Each of these keywords holds the intervals of date in which the bursts occurred. All 71 keywords cover the research frontier of mHealth to a great extent. In addition, the frequencies of these keywords are 2028 times, showing that 1.28% (71/5543) of keywords accounted for 16.46% (2028/12,318) of the total 12,318 frequencies.

**Figure 7:**
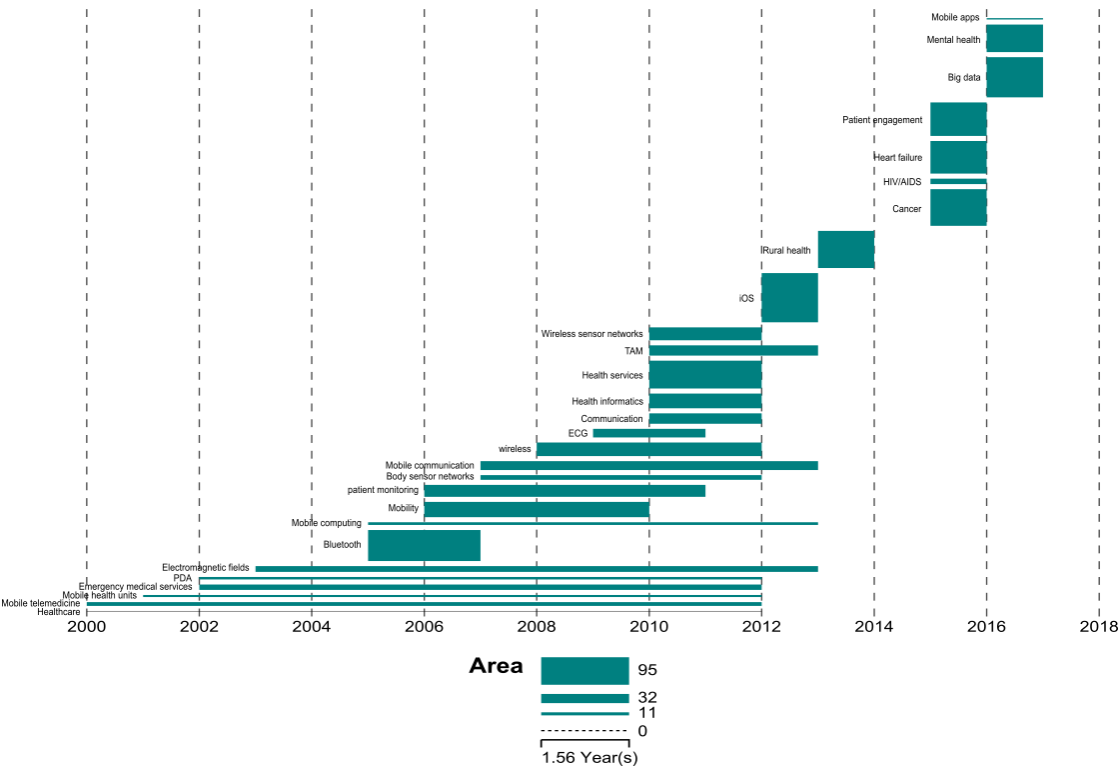
Temporal bar graph for burst terms. ECG: electrocardiogram; PDA: personal digital assistant; TAM: technology acceptance model.

The temporal bar graph for the 27 burst terms clearly represents an evolution in topics over time, demonstrating the updating and interacting of the literature. In Figure 7, we can see that mobile telemedicine, mHealth units, and PDA were run through the research on mHealth in the period between 2000 and 2012, suggesting that the application of mobile technologies in health care has begun to receive greater attention. This phenomenon corresponds to the widespread application of information technology in every walk of life in the early 21st century. In this body of knowledge, we can identify some main devices that have been applied to the health care field during the period of 2005 to 2010, including Bluetooth, body sensor networks, and mobile computing.

From 2010 to 2014, the major burst terms were technology acceptance model (TAM), iOS, health services, and rural health. It showed that the research focus had turned to the integration of health technology with health services, and that researchers had begun to explore how to improve technological acceptation from users. The representative burst terms from 2015 to 2016 were patient engagement, mental health, illness, and big data, suggesting that patient engagement and smart prevention methods have been a major research focus in the current new technology environment in the recent years.

#### Research Topic Distribution

The 71 keywords identified in the mHealth field were divided into 12 clusters through hierarchical cluster analysis, indicating the topics are broad and varied. The cluster name of each cluster was refined, based on the keywords in the respective cluster, all of which are presented in Table 5. That is, Cluster 1 refers to security and privacy; Cluster 2 focuses on health monitoring and u-health; Cluster 3 is associated to health care and mobile computing; Cluster 4 is related to body sensor networks and patient monitoring; Cluster 5 refers to cell phones and health surveillance; Cluster 6 is about text messaging and health intervention; Cluster 7 focuses on social support, social media, and health promotion; Cluster 8 is related to mobile apps and mental health; Cluster 9 refers to mobile technology, nursing, and data mining; Cluster 10 is associated to self-care and patient engagement; Cluster 11 focuses on health services and health education; and Cluster 12 is related to TAM, chronic disease, and home health monitoring.

#### Social Network Analysis

**Figure 8:**
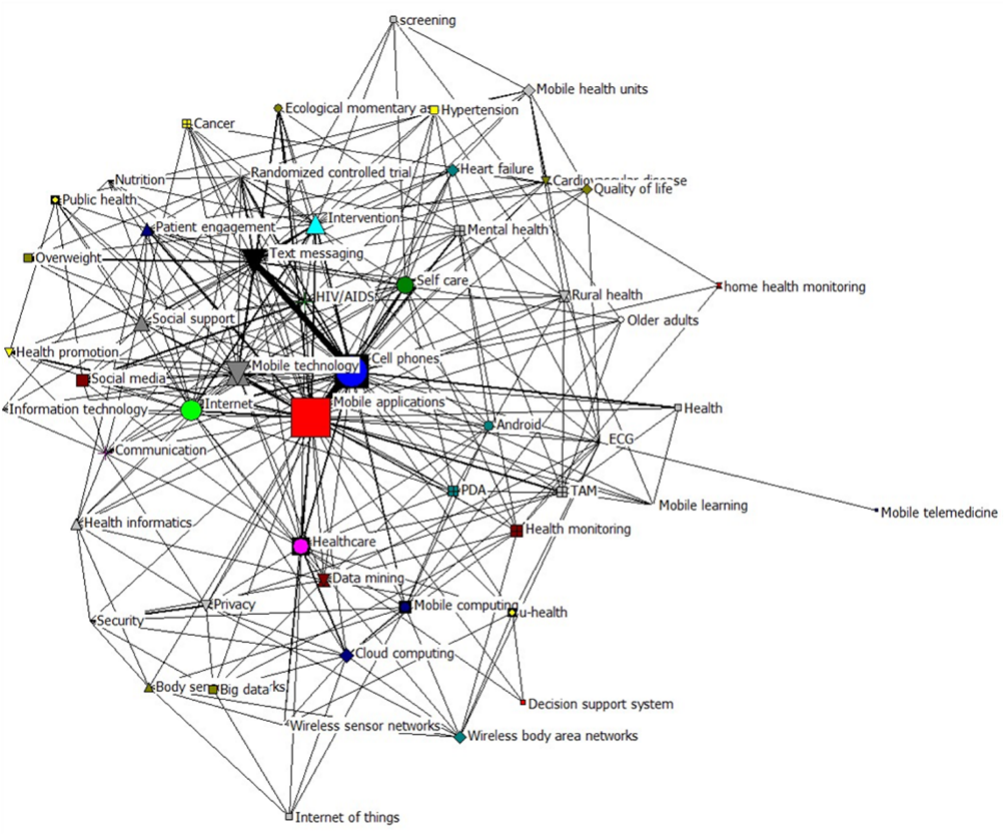
Social network map of the original 52 × 52 co-occurrence matrix. ECG: electrocardiogram; PDA: personal digital assistant; TAM: technology acceptance model.

On the basis of the 71×71 similarity matrix, it was possible to calculate the density of the network, which is 0.007, a relatively low level. To explicitly demonstrate the networking relationship and obtain more powerful and intuitive results, we formed the 52×52 co-occurrence matrix based on the original 71×71 co-occurrence matrix, of which the keyword that correspond to any row or any column has not less than 12 frequencies. On the basis of the new matrix, a network was generated using Netdraw2.0 embedded in Ucinet6.6 (shown in Figure 8), which intuitively reflects the relationships among the high-frequency and highly bursting keywords.

The graph shown in Figure 8 semantically interrelates and chronologically links diverse fields of mHealth research. A total of 4 major areas can be identified: (1) the top left subnetwork is related to patient engagement and patient intervention research, which mostly covers Clusters 5-7; (2) the top right topics deal with health monitoring and self-care research, which roughly include Cluster 2, Cluster 8, Cluster 10, and Cluster 12; (3) the bottom right is linked to mobile device and mobile computing research, which mainly contains Clusters 3-4 and Cluster 9; and (4) the bottom left relates to security and privacy studies, which includes Cluster 1.

**Table 5:** Twelve clusters of mobile health (mHealth) research.

Cluster	Number of keywords	Cluster name	Keywords
1	2	Security and privacy	Security; privacy
2	8	Health monitoring and u-health	ECG^a^; cloud computing; wireless body area networks; health monitoring; big data; mobile telemedicine; u-health; wireless
3	4	Health care and mobile computing	Health care; mobile computing; Internet of things; ubiquitous computing
4	4	Body sensor networks and patient monitoring	Body sensor networks; wireless sensor networks; decision support system; patient monitoring; mobility; Bluetooth
5	6	Cell phones and health surveillance	Cell phones; health; surveillance; epidemiology; informatics; electromagnetic fields; maternal health
6	7	Text messaging and health intervention	Text messaging; HIV/AIDS; randomized controlled trial; cancer; overweight; nutrition; intervention study
7	7	Social support, social media and health promotion	Internet; intervention; social support; social media; health promotion; communication; public health
8	4	Mobile apps and mental health	Mobile apps; ecological momentary assessment; mental health; bipolar disorder
9	7	Mobile technology, nursing, and data mining	Mobile technology; PDA^b^; health informatics; Android; data mining; nursing; iOS
10	4	Self-care and patient engagement	Self-care; patient engagement; heart failure; quality of life
11	4	Health services and health education	Information technology; health services; emergency medical services; health education
12	11	TAM^c^, chronic disease, and home health monitoring	Mobile health units; cardiovascular disease; TAM; rural health; home health monitoring; older adults; hypertension; mobile learning; screening; implementation; mobile communication

^a^ECG: electrocardiogram.

^b^PDA: personal digital assistant.

^c^TAM: technology acceptance model.

## Discussion

### Principal Findings

This study has demonstrated that the growth of literature related to mHealth research has accelerated in recent years, and that the influence is continually increasing. As we know, with the emergence of mobile phone technologies from 2006 to 2010, the field of mHealth entered a phase of rapid innovation and, in parallel, unfettered proliferation. In addition, mHealth Alliance, founded in 2008, has played an important role in advancing the field of mHealth through thought leadership and by convening a range of stakeholders, which focuses on advancing mHealth technology in global health care through policy research, advocacy, and outreach [50]. Obviously, all of these events present a large research scope on mHealth, and the number of publications started to increase dramatically during the period from 2009 to 2016, although it was less before 2008. In addition, based on the logical growth curve equation of mHealth literature, combined with the fact that some important papers published before December 28, 2016, are not indexed in WoSCC, it is reasonable to infer that mHealth literature published in 2017 is expected to reach more than 1000, and that the average annual growth rate of literature on mHealth will reach nearly 60% (648/410-1) after 2016.

Additionally, IF is seen to be a metric of excellence for journals, that is, journals with higher IF are often deemed to be more important than those with lower IF. In the past 2 years, there is an increase of IF for major journals related to mHealth, which further reflects the concern of researchers to mHealth.

Our study showed that the degree of author collaboration is relatively high, that the core author group is fundamentally formed, and that collaboration between the core authors should be further strengthened. Scientific collaboration has become prevalent in various disciplines [51]. In this study, the total degree of author collaboration was more than 4 during the past 2 decades and, compared with other disciplines, the collaboration degree of international mHealth stays above the average level in scientific collaboration [52]. As we understand, with the development of science and the explosion of knowledge, nobody is an expert in everything [53]. It is an arduous task for an individual to fully understand the extensive knowledge of various fields in the information era. Research on mHealth is no exception and this requires more experts from different field to collaborate together to answer important questions. Additionally, collaboration can facilitate the sharing and dissemination of knowledge and attract more attention to the field [54,55].

Obviously, some specific authors played very important roles and had a big impact in the mHealth field and on future development, representing “core strength” in this field. The output of core authors in the mHealth field represents approximately 50% (1435/2704) of the total number of publications. According to the Law of Price, we can find that the core authors group has fundamentally been formed and that the publication output of the core authors will increase over time. However, there are only 4 major clusters of authors, which can be regarded as the backbone in the field, indicating that the current collaboration of core authors is not tight and stable. Moreover, when combined with the analysis above, it can be concluded that the core researchers in the mHealth field should further strengthen their collaboration to form a more stable and core collaborative group.

In our study, it can be seen that the leading research power is in the United States and that the collaborative relationship of institutions or countries is not relatively tight. Although 3040 institutions have been involved in research on mHealth, indicating a remarkable concern, publication output on mHealth research is distributed unevenly between institutions. Links between institutions are relatively few, according to the collaborative relationship map, which means less collaboration among institutions and less willingness to collaborate, except for the network consisting of numerous American colleges and universities. Additionally, it can be inferred that universities are major research forces, similar to other research fields. Institution collaboration, in general, should be further strengthened in future. Moreover, combining the sparse institution collaboration, it can be further inferred that the collaboration mainly occurs among authors with different academic professional backgrounds from the same institution.

Furthermore, the major industrialized countries (such as G7 countries: the United States, the United Kingdom, Germany, Canada, Italy, France, and Japan) are mostly in the core of the country collaboration network, suggesting that economic development and scientific investment have much contribution to the publication outputs in mHealth. However, China, which is representative of the developing countries, also pays more attention to public health and plays a prior role in the mHealth studies. Additionally, based on the country collaboration graph, it can be inferred that institutions located in the United States are more inclined to collaborate with domestic institutions, suggesting institutions in the United States have a relatively low tendency toward international collaboration. In fact, scientific collaboration relationships are highly resource-dependent [56] and internationalization of science, to a certain degree, depends on the attractiveness of a partner in the global network. Therefore, the international collaboration for institutions in mHealth research encounters challenges as well, particularly for developing countries that are confronted with critical internal conditions (eg, policy and funds) that often prevent them from collaborating with high scientific capacity. To change this situation, measures should be taken which will benefit the developing country itself from the application of mHealth in the near future. For example, more scholars related to mHealth research from the developing country should be supported by related countries or institutions to study and communicate in the United States, or some advanced experts could be invited to guide the research in the developing country.

In this study, we find that modern ICT is increasingly being integrated with health care systems, and that research topic burst on mHealth is relatively decentralized. The 71 identified keywords demonstrate the research frontier of mHealth field to a very great extent. From the temporal bar graph, it can be seen that research focus has already begun to shift from acceptance and feasibility to outcome of mHealth to some extent and that patient engagement through social media and mobile computing has started receiving more attention in recent years. Generally, mHealth has been seen to be a new effective approach to increasing means and efficiency of care delivery in the health domain using ICT. Additionally, mHealth would provide support for medical service decisions by means of second development and utilization of medical and health care data, such as mobile computing and data mining.

The hierarchical cluster analysis intuitively displays the 12 keyword clusters and the relationship between topics, each of which represents a research direction on mHealth. Compared with other studies, mHealth research topics are relatively decentralized at present, and social network analysis presents the 4 major areas, each of which covers 1 or several of 12 clusters. In addition, the top left subnetwork receives more attention than others. Generally, research on mHealth should be further strengthened in these 4 areas, and the research topics would also need to be further focused in future, which will be beneficial to health care services.

### Limitations

Although findings are based on the above analysis, there are still several potential limitations that may encourage further research efforts. First, this study only focuses on literature indexed by WoSCC. Although WoSCC emphases paper quality to ensure accurate and meaningful data, it leads to some papers related to mHealth not being covered. Moreover, there are several high-quality papers that are still not indexed by WoSCC due to time-lag, especially those published at the end of 2016. All of these will have some impact on the accuracy of research output on mHealth.

Second, there might be some biases of understanding for author collaboration because some different authors with the same name or abbreviation exist, who are affiliated to different institutions. In addition, some authors are simply “token co-authors” included in some papers. Therefore, the result of author relationship analysis for mHealth research would be influenced by the accuracy of the indexing author.

Finally, although temporal analysis and hierarchy cluster analysis are quite useful methods for exploring topic evolution and identifying hotspots in 1 field, the results may be affected by the accuracy of keywords. We used 3 main methods of cleaning keywords in this research, but there still exists some keywords with the same meaning, which will affect the cluster results to some extent.

### Conclusions

In this study, a comprehensive bibliometric analysis on mHealth research was conducted, with the data source being the WoSCC, using various tools. Different visualization methods were used to interactively explore and understand the specific datasets. On the basis of the above results and discussion, some valuable results for mHealth research were obtained, including information on collaboration characteristics and research topic bursts. Meanwhile, with the deep contingency of mobile technologies and health care services, it is reasonable to believe that the literature related to mHealth research will grow at an exponential rate in future and that the collaboration of core authors will strengthen after core author groups officially form. In addition, although the United States has the leading research power in mHealth area, the collaborative relationship of institutions or countries should be reinforced to promote the global mHealth field. In general, the focus of research topics on mHealth should be enhanced in the future.

It should be noted that mHealth has begun to be an important part of digital health, which is the convergence of digital and genomic technologies with health, health care, living, and society to enhance the efficiency of health care delivery and make medicines more personalized and precise [57]. The broad scope of digital health includes categories such as mHealth, health information technology, wearable devices, telehealth and telemedicine, and personalized medicine [58-60]. All of these are helpful to promote the emergence and development of quality research and provide a potential guideline for scientific researchers when launching new projects in the future.

## Appendix

Multimedia Appendix 1 Detailed search strategy of WoSCC.

Multimedia Appendix 2 List of papers published as top 7 most productive first authors.

Multimedia Appendix 3 List of relevant information for main authors of the 4 clusters in collaboration relationship map of productive authors.

Multimedia Appendix 4 The equation for the minimum output of core author.

Multimedia Appendix 5 List of the corresponding relations between the abbreviations and the full forms of the main institutions.

Multimedia Appendix 6 List of the corresponding relations between the abbreviations and the full forms of the main countries and territories.

Multimedia Appendix 7 List of keywords with burst strength more than 1 and frequency not less than 10.

## Abbreviations

AGCS: average global citation score
ALCS: average local citation score
BICOMS: Bibliographic Item Co-Occurrence Mining System
ECG: electrocardiogram
ICT: information and communication technologies
IF: impact factor
LMICs: lower middle-income countries
mHealth: mobile health
PDA: personal digital assistant
Sci2: Science of Science
SMS: short messaging service
TAM: technology acceptance model
TGCS: total global citation score
TLCS: total local citation score
WoSCC: Web of Science Core Collection
