# Supplementary material for: Visualizing Collaboration Characteristics and Topic Burst on International Mobile Health Research: Bibliometric Analysis
Source: JMIR Mhealth Uhealth. 2018 Jun 5;6(6):e135. doi: 10.2196/mhealth.9581 (PMC6008511; doi:10.2196/mhealth.9581)
Supplement: Multimedia Appendix 1 [file mhealth_v6i6e135_app1.pdf]

Detailed search strategy of WoSCC in this study as below:

#1 mobile health

Search details of WoSCC: TS = ("m$Health*" OR "m Health*" OR "mcare" OR "mobile health*" OR "mobile$care" OR "mobile care" OR "mobile medical" OR "mobile teleMedicine" OR "mobile tele$health*" OR "mobile e$health*")

#2 mHealth apps

Search details of WoSCC: TI = ("m$health app*" OR "health* app" OR "health* apps") OR (("mobile app*" OR "web app*" OR "cloud app*") AND ("health*" OR "care" OR "medical"))

#3 TS = (("mobile technolog*" OR "mobile device*") AND "health*")

#4 TI = (("mobile phone*" OR "tablet comput*" OR "personal digital assistant*") AND "health*")

#5 TI = ("mobile unit*" OR TI="mobile health unit*")

#6 (#1 OR #2 OR #3 OR #4) NOT #5.

(In addition, it should be noted that the field tag "TS" refers to "Topic", whereas the field tag "TI" means "Title".)
