# Supplementary material for: Visualizing Collaboration Characteristics and Topic Burst on International Mobile Health Research: Bibliometric Analysis
Source: JMIR Mhealth Uhealth. 2018 Jun 5;6(6):e135. doi: 10.2196/mhealth.9581 (PMC6008511; doi:10.2196/mhealth.9581)
Supplement: Multimedia Appendix 2 [file mhealth_v6i6e135_app2.pdf]

List of papers published as top7 most productive first authors

1. John D. Piette

[1] Piette, J. D., Mendozaavelares, M. O., Milton, E. C., Lange, I., & Fajardo, R. (2010). Access to mobile communication technology and willingness to participate in automated telemedicine calls among chronically ill patients in honduras. Telemedicine Journal and e-Health, 16(10), 1030. [PMID: 21062234].

[2] Piette, J. D., Datwani, H., Gaudioso, S., Foster, S. M., Westphal, J., & Perry, W., et al. (2012). Hypertension management using mobile technology and home blood pressure monitoring: results of a randomized trial in two low/middle-income countries. Telemedicine journal and e-health : the official journal of the American Telemedicine Association, 18(8), 613-20. [PMID: 23061642].

[3] Piette JD1, RoslandAM, Marinec NS, Striplin D, Bernstein SJ, Silveira MJ. Engagement with automated patient monitoring and self-management support calls: experience with a thousand chronically ill patients. 2013,51(3):216-23. [PMID: 23222527].

[4] Piette, J. D., Sussman, J. B., Pfeiffer, P. N., Silveira, M. J., Singh, S., & Lavieri, M. S. (2013). Maximizing the value of mobile health monitoring by avoiding redundant patient reports: prediction of depression-related symptoms and adherence problems in automated health assessment services.Journal of Medical Internet Research, 15(7), e118. [PMID: 23832021].

[5] Piette, J. D., Aikens, J. E., Rosland, A. M., & Sussman, J. B. (2014). Rethinking the frequency of between-visit monitoring for patients with diabetes. Medical Care, 52(6), 511-8. [PMID: 24824536].

[6] Piette, J. D., Farris, K. B., Newman, S., An, L., Sussman, J., & Singh, S. (2015). The potential impact of intelligent systems for mobile health self-management support: monte carlo simulations of text message support for medication adherence. Annals of Behavioral Medicine, 49(1), 84-94. [PMID: 25082177].

[7] Piette, J. D., Striplin, D., Marinec, N., Chen, J., Trivedi, R. B., & Aron, D. C., et al. (2015). A mobile health intervention supporting heart failure patients and their informal caregivers: a randomized comparative effectiveness trial. Journal of Medical Internet Research, 17(6), e142. [PMID: 26063161].

[8] Piette, J. D., Striplin, D., Marinec, N., Chen, J., & Aikens, J. E. (2015). A randomized trial of mobile health support for heart failure patients and their informal caregivers: impacts on caregiver-reported outcomes. Medical Care, 53(8), 692. [PMID: 26125415].

[9] Piette, J. D., List, J., Rana, G. K., Townsend, W., Striplin, D., &Heisler, M. (2015). Mobile health

devices as tools for worldwide cardiovascular risk reduction and disease management.Circulation, 132(21), 2012. [PMID: 26596977].

[10] Piette, J. D., Marinec, N., Janda, K., Morgan, E., Schantz, K., & Yujra, A. C., et al. (2016). Structured caregiver feedback enhances engagement and impact of mobile health support: a randomized trial in a lower-middle-income country. Telemed J E Health, 22(4), 261-268. [PMID: 26352854].

[11] Piette, J. D., Krein, S. L., Striplin, D., Marinec, N., Kerns, R. D., & Farris, K. B., et al. (2016). Patient-centered pain care using artificial intelligence and mobile health tools: protocol for a randomized study funded by the us department of veterans affairs health services research and development program. Jmir Research Protocols, 5(2), e53. [PMID: 27056770].

2. Dror Ben-Zeev

[1] Ben-Zeev, D., Frounfelker, R., Morris, S. B., & Corrigan, P. W. (2012). Predictors of self-stigma in schizophrenia: new insights using mobile technologies. Journal of Dual Diagnosis, 8(4), 305-314. [PMID: 23459025].

[2] Ben-Zeev, D., Mchugo, G. J., Xie, H., Dobbins, K., & Young, M. A. (2012). Comparing retrospective reports to real-time/real-place mobile assessments in individuals with schizophrenia and a nonclinical comparison group. Schizophrenia Bulletin, 38(3), 396-404. [PMID: 22302902].

[3] Ben-Zeev, D., Davis, K. E., Kaiser, S., Krzsos, I., & Drake, R. E. (2013). Mobile technologies among people with serious mental illness: opportunities for future services. Administration and Policy in Mental Health and Mental Health Services Research, 40(4), 340-343. [PMID: 22648635].

[4] Ben-Zeev, D., Kaiser, S. M., Brenner, C. J., Begale, M., Duffecy, J., & Mohr, D. C. (2013). Development and usability testing of focus: a smartphone system for self-management of schizophrenia. Psychiatric Rehabilitation Journal, 36(4), 289.[PMID: 24015913].

[5] Ben-Zeev, D., Kaiser, S. M., & Krzos, I. (2014). Remote "hovering" with individuals with psychotic disorders and substance use: feasibility, engagement, and therapeutic alliance with a text-messaging mobile interventionist. Journal of Dual Diagnosis, 10(4), 197-203. [PMID: 25391277]

[6]Ben-Zeev, D., Brenner, C. J., Begale, M., Duffecy, J., Mohr, D. C., & Mueser, K. T. (2014). Feasibility, acceptability, and preliminary efficacy of a smartphone intervention for schizophrenia. Schizophrenia Bulletin, 40(6), 1244-53. [PMID: 24609454].

[7] Ben-Zeev, D., Schueller, S. M., Begale, M., Duffecy, J., Kane, J. M., & Mohr, D. C. (2015). Strategies for mhealth research: lessons from 3 mobile intervention studies. Administration &

Policy in Mental Health & Mental Health Services Research, 42(2), 157-167. [PMID: 24824311].

[8] Ben-Zeev, D., Scherer, E. A., Wang, R., Xie, H., & Campbell, A. T. (2015). Next-generation psychiatric assessment: using smartphone sensors to monitor behavior and mental health. PsychiatrRehabil J, 38(3), 218-226.[PMID: 26691997].

3. David D.Luxton

[1] Luxton, D. D., June, J. D., & Kinn, J. T. (2011). Technology-based suicide prevention: current applications and future directions. Telemedicine & e-Health, 17(1), 50.[PMID: 21214382].

[2] Luxton, D. D., Mccann, R. A., Bush, N. E., Mishkind, M. C., & Reger, G. M. (2011). Mhealth for mental health: integrating smartphone technology in behavioral healthcare. Professional Psychology Research & Practice, 42(6), 505-512.

[3] Luxton, D. D., Kayl, R. A., & Mishkind, M. C. (2012). Mhealth data security: the need for hipaa-compliant standardization. Telemedicine journal and e-health : the official journal of the American Telemedicine Association, 18(4), 284.[PMID: 22400974].

[4] Luxton, D. D., Mishkind, M. C., Crumpton, R. M., Ayers, T. D., & Mysliwiec, V. (2012). Usability and feasibility of smartphone video capabilities for telehealth care in the u.s. military. *Telemedicine journal and e-health : the official journal of the American Telemedicine Association*, *18*(6), 409.[PMID: 22650351].

[5] Luxton, D. D., Pruitt, L. D., & Osenbach, J. E. (2014). Best practices for remote psychological assessment via telehealth technologies. Professional Psychology Research & Practice, 45(1), 27-35.

[6] Luxton, D. D., Hansen, R. N., & Stanfill, K. (2014). Mobile app self-care versus in-office care for stress reduction: a cost minimization analysis. Journal of Telemedicine & Telecare, 20(8), 431-435.[PMID: 25316037].

4. Arul Chib

[1] Chib, A. (2010). The aceh besar midwives with mobile phones project: design and evaluation perspectives using the information and communication technologies for healthcare development model. Journal of Computer‐mediated Communication, 15(3), 500-525.

[2] Chib, A., & Chen, H. H. (2011). Midwives with mobiles: a dialectical perspective on gender arising from technology introduction in rural indonesia. New Media & Society, 13(3), 486-501.

[3] Chib, A., Wilkin, H., Ling, L. X., Hoefman, B., & Van, B. H. (2012). You have an important

message! evaluating the effectiveness of a text message hiv/aids campaign in northwest uganda. J Health Commun, 17(sup1), 146-157. [PMID: 22548607]

[4] Arul Chib, Tran Khanh Phuong, Chia Wei Si, & Ng Su Hway. (2013). Enabling informal digital guanxi for rural doctors in shaanxi, china. Chinese Journal of Communication, 6(1), 62-80.

[5] Chib, A., Wilkin, H., & Hoefman, B. (2013). Vulnerabilities in mhealth implementation: a ugandan hiv/aids sms campaign. Global Health Promotion, 20(1 Suppl), 26. [PMID: 23549699]

5. Gabrielle Turner-McGrievy

[1] Turner-Mcgrievy, G. M., Beets, M. W., Moore, J. B., Kaczynski, A. T., Barranderson, D. J., & Tate, D. F. (2013). Comparison of traditional versus mobile app self-monitoring of physical activity and dietary intake among overweight adults participating in an mhealth weight loss program. Journal of the American Medical Informatics Association Jamia, 20(3), 513. [PMID: 23429637]

[2] Turner-Mcgrievy, G. M., & Tate, D. F. (2013). Weight loss social support in 140 characters or less: use of an online social network in a remotely delivered weight loss intervention. Transl Behav Med, 3(3), 287-94. [PMID: 24073180]

[3] Turner-Mcgrievy, G. M., Davidson, C. R., & Wilcox, S. (2014). Does the type of weight loss diet affect who participates in a behavioral weight loss intervention? a comparison of participants for a plant-based diet versus a standard diet trial. Appetite, 73(2), 156-162. [PMID: 24269507]

[4] Turner-Mcgrievy, G. M., & Tate, D. F. (2014). Are we sure that mobile health is really mobile? an examination of mobile device use during two remotely-delivered weight loss interventions. International Journal of Medical Informatics, 83(5), 313-319. [PMID: 24556530]

[5] Turner-Mcgrievy, G. M., Helander, E. E., Kaipainen, K., Perez-Macias, J. M., & Korhonen, I. (2015). The use of crowdsourcing for dietary self-monitoring: crowdsourced ratings of food pictures are comparable to ratings by trained observers. J Am Med Inform Assoc, 22(e1), e112-e119. [PMID: 25092793]

6. Kelly A. Aschbrenner

[1] Aschbrenner, K. A., Naslund, J. A., Barre, L. K., Mueser, K. T., Kinney, A., & Bartels, S. J. (2015). Peer health coaching for overweight and obese individuals with serious mental illness: intervention development and initial feasibility study. Transl Behav Med, 5(3), 277-284. [PMID: 26327933]

[2] Aschbrenner, K. A., Naslund, J. A., Gill, L. E., Bartels, S. J., & Ben-Zeev, D. (2016). A qualitative study of client-clinician text exchanges in a mobile health intervention for individuals

with psychotic disorders and substance use. Journal of Dual Diagnosis, 12(1), 00-00. [PMID: 26829356]

[3] Aschbrenner, K. A., Naslund, J. A., Shevenell, M., Kinney, E., & Bartels, S. J. (2016). A pilot study of a peer-group lifestyle intervention enhanced with mhealth technology and social media for adults with serious mental illness. Journal of Nervous & Mental Disease, 204(6), 483-486. [PMID: 27233056]

[4] Aschbrenner, K. A., Naslund, J. A., Shevenell, M., Mueser, K. T., & Bartels, S. J. (2016). Feasibility of behavioral weight loss treatment enhanced with peer support and mobile health technology for individuals with serious mental illness. Psychiatric Quarterly, 87(3), 1-15. [PMID: 26462674]

[5] Aschbrenner, K. A., Naslund, J. A., & Bartels, S. J. (2016). A mixed methods study of peer-to-peer support in a group-based lifestyle intervention for adults with serious mental illness. Psychiatr Rehabil J, 39(4), 328-334. [PMID: 27560454]

7. Shahriar Akter

[1] Akter, S., D’Ambra, J., & Ray, P. (2010). Service quality of mhealth platforms: development and validation of a hierarchical model using pls. Electronic Markets, 20(3-4), 209-227.

[2] Akter, S., D'Ambra, J., & Ray, P. (2011). Trustworthiness in mhealth information services: an assessment of a hierarchical model with mediating and moderating effects using partial least squares (pls). Journal of the Association for Information Science & Technology, 62(1), 100-116.

[3] Akter, S., Ray, P., & D’Ambra, J. (2013). Continuance of mhealth services at the bottom of the pyramid: the roles of service quality and trust. Electronic Markets, 23(1), 29-47.

[4] Akter, S., D’Ambra, J., & Ray, P. (2013). Development and validation of an instrument to measure user perceived service quality of mHealth. Elsevier Science Publishers B. V, 50 (4), 181-195.

[5] Akter, S., D'Ambra, J., Ray, P., & Hani, U. (2012). Modelling the Impact of mHealth Service Quality on Satisfaction, Continuance and Quality of Life. Behaviour & Information Technology, 32(12), 1225-1241.
