# Supplementary material for: Visualizing Collaboration Characteristics and Topic Burst on International Mobile Health Research: Bibliometric Analysis
Source: JMIR Mhealth Uhealth. 2018 Jun 5;6(6):e135. doi: 10.2196/mhealth.9581 (PMC6008511; doi:10.2196/mhealth.9581)
Supplement: Multimedia Appendix 3 [file mhealth_v6i6e135_app3.pdf]

List of relevant information for main authors of 4 clusters in collaborative relationship map of productive authors.

Cluster 1:

No.	Author name (full name)	Main affiliation	Counry
1	Piette JD (John D. Piette)	Ann Arbor Department of VA Center for Clinical Management Research, Michigan	USA
2	Allman-Farinelli M (M. Allman-Farinelli)	University of Sydney, Sydney	Australia
3	Bauman A (Adrian Bauman)	University of Sydney, Sydney	Australia
4	Aikens JE (James E. Aikens)	University of Michigan, Ann Arbor, MI	USA
5	Chen J (Juliana Chen)	University of Sydney, Sydney	Australia

Cluster 2:

No.	Author name (full name)	Main affiliation	Country
1	Whittaker R (Robyn Whittaker)	University of Auckland, Auckland	New Zealand
2	Maddison R (Ralph Maddison)	University of Auckland, Auckland	New Zealand
3	Jiang YN (Yannan Jiang)	University of Auckland, Auckland	New Zealand

Cluster 3:

No.	Author name (full name)	Main affiliation	Country
1	Aschbrenner KA (Kelly A. Aschbrenner)	Geisel School of Medicine at Dartmouth, Lebanon, NH	USA
2	Naslund JA (John A. Naslund)	Geisel School of Medicine at Dartmouth, Lebanon, NH	USA
3	Bartels SJ (Stephen J. Bartels)	Geisel School of Medicine at Dartmouth, Lebanon, NH	USA

Cluster 4:

No.	Author name<br>(full name)	Main affiliation	Country
1	Wang W<br>(Wei Wang)	Capital Institute of Pediatrics, Beijing	China
2	Wu Q<br>(Qiong Wu)	Capital Institute of Pediatrics, Beijing	China
3	Chen L<br>(Li Chen)	Capital Institute of Pediatrics, Beijing	China
4	Li Y<br>(Ye Li)	Capital Institute of Pediatrics, Beijing	China
