# Supplementary material for: Visualizing Collaboration Characteristics and Topic Burst on International Mobile Health Research: Bibliometric Analysis
Source: JMIR Mhealth Uhealth. 2018 Jun 5;6(6):e135. doi: 10.2196/mhealth.9581 (PMC6008511; doi:10.2196/mhealth.9581)
Supplement: Multimedia Appendix 4 [file mhealth_v6i6e135_app4.pdf]

The equation for the minimum output of core author

The equation for the minimum output of core author is $M \cong 0.749(N_{\max})^{1/2}$, where M is the minimum output of core author and $N_{\max}$ is the maximum output of core author.
