# Supplementary material for: Visualizing Collaboration Characteristics and Topic Burst on International Mobile Health Research: Bibliometric Analysis
Source: JMIR Mhealth Uhealth. 2018 Jun 5;6(6):e135. doi: 10.2196/mhealth.9581 (PMC6008511; doi:10.2196/mhealth.9581)
Supplement: Multimedia Appendix 5 [file mhealth_v6i6e135_app5.pdf]

List of the corresponding relations between the abbreviations and the full forms of the main institutions.

No.	Abbreviation	Full form
1	Vanderbilt Univ	Vanderbilt University
2	Dartmouth Coll	Dartmouth College
3	Univ Pittsburgh	University of Pittsburgh
4	Univ Calif San Francisco	University of California, San Francisco
5	Univ Michigan	University of Michigan
6	Univ Penn	University of Pennsylvania
7	Northwestern Univ	Northwestern University
8	Johns Hopkins Bloomberg Sch Publ Hlth	Johns Hopkins Bloomberg School of Public Health
9	Harvard Univ	Harvard University
10	Univ Washington	University of Washington
11	Univ Calif Los Angeles	University of California, Los Angeles
12	Stanford Univ	Stanford University
13	Columbia Univ	Columbia University
14	Duke Univ	Duke University
15	Univ Calif San Diego	University of California, San Diego
16	Univ British Columbia	University of British Columbia
