# Supplementary material for: Visualizing Collaboration Characteristics and Topic Burst on International Mobile Health Research: Bibliometric Analysis
Source: JMIR Mhealth Uhealth. 2018 Jun 5;6(6):e135. doi: 10.2196/mhealth.9581 (PMC6008511; doi:10.2196/mhealth.9581)
Supplement: Multimedia Appendix 6 [file mhealth_v6i6e135_app6.pdf]

List of the corresponding relations between the abbreviations and the full forms of the main countries and territories.

No.	Abbreviation	Full name
1	USA	United States
2	PEOPLES R CHINA	People's Republic of China
3	ENGLAND	England
4	AUSTRALIA	Australia
5	CANADA	Canada
6	SOUTH KOREA	South Korea
7	TAIWAN	Taiwan
8	SPAIN	Spain
9	GERMANY	Germany
10	NETHERLANDS	Netherlands
11	ITALY	Italy
12	INDIA	India
13	FRANCE	France
14	SINGAPORE	Singapore
15	JAPAN	Japan
16	SWEDEN	Sweden
17	GREECE	Greece
18	BELGIUM	Belgium
19	FINLAND	Finland
20	POLAND	Poland
21	DENMARK	Denmark
