# Supplementary material for: Visualizing Collaboration Characteristics and Topic Burst on International Mobile Health Research: Bibliometric Analysis
Source: JMIR Mhealth Uhealth. 2018 Jun 5;6(6):e135. doi: 10.2196/mhealth.9581 (PMC6008511; doi:10.2196/mhealth.9581)
Supplement: Multimedia Appendix 7 [file mhealth_v6i6e135_app7.pdf]

List of keywords with burst strength >1 and frequency ≥10.

No.	Keyword	Frequency	Burst strength	Start date-end date	No.	Keyword	Frequency	Burst strength	Start date-end date	No.	Keyword	Frequency	Burst strength	Start date-end date
**1**	Mobile apps	252	4.20	2016-2016	**25**	Mental health	19	2.64	2016-2016	**49**	Mobile telemedicine	12	5.96	2000-2012
**2**	Cell phones	230	1.39	2011-2011	**26**	Wireless sensor networks	19	2.42	2010-2012	**50**	Nutrition	12	1.95	2016-2016
**3**	Text messaging	166	1.43	2013-2013	**27**	Cardiovascular disease	19	1.63	2012-2012	**51**	u-health	12	1.23	2009-2013
**4**	Mobile technology	104	1.49	2006-2007	**28**	Health informatics	18	3.25	2010-2012	**52**	Data mining	12	1.19	2003-2011
**5**	Health care	63	4.92	2000-2012	**29**	Health	18	1.07	2013-2013	**53**	Mobile communication	11	3.30	2007-2013
**6**	HIV/AIDS	62	2.38	2015-2016	**30**	TAM[a]	17	3.14	2010-2013	**54**	Wireless	11	3.07	2008-2012
**7**	Internet	60	1.99	2000-2006	**31**	Heart failure	17	2.65	2015-2016	**55**	Emergency medical services	11	2.71	2002-2012
**8**	Self-care	56	1.80	2015-2016	**32**	Rural health	17	1.87	2008-2013	**56**	iOS	11	2.46	2012-2013
**9**	Intervention	35	1.24	2005-2005	**33**	Android	17	1.59	2012-2016	**57**	Health services	11	2.05	2010-2012
**10**	Security	31	1.89	2008-2012	**34**	Wireless body area networks	17	1.27	2010-2012	**58**	Ubiquitous computing	11	1.76	2012-2013
**11**	Social support	31	1.33	2013-2013	**35**	Internet of things	17	1.25	2015-2016	**59**	Surveillance	11	1.76	2012-2013

**12**	Social media	28	1.31	2016-2016	**36**	Information technology	16	1.28	2009-2010	**60**	Bipolar disorder	11	1.56	2016-2016
**13**	Patient engagement	26	2.97	2015-2016	**37**	Home health monitoring	16	1.27	2010-2013	**61**	Health education	11	1.47	2013-2014
**14**	ECG[b]	25	3.39	2009-2011	**38**	Older adults	16	1.08	2014-2014	**62**	Nursing	11	1.16	2006-2010
**15**	Randomized controlled trial	25	1.31	2015-2016	**39**	Public health	15	1.43	2001-2011	**63**	Implementation	11	1.04	2013-2013
**16**	Ecological momentary assessment	25	1.26	2012-2012	**40**	Hypertension	15	1.06	2015-2016	**64**	Electromagnetic fields	10	2.67	2003-2013
**17**	Health promotion	25	1.12	2014-2014	**41**	Cancer	14	2.01	2015-2016	**65**	Mobility	10	2.64	2006-2010
**18**	Privacy	24	1.36	2010-2010	**42**	Health monitoring	14	1.38	2009-2010	**66**	Bluetooth	10	2.54	2005-2007
**19**	Communication	23	2.60	2010-2012	**43**	Mobile learning	14	1.35	2008-2011	**67**	Patient monitoring	10	2.26	2006-2011
**20**	Mobile computing	23	2.52	2005-2013	**44**	Big data	13	2.35	2016-2016	**68**	Informatics	10	1.91	2012-2013
**21**	Mobile health units	22	5.42	2001-2012	**45**	Overweight	13	1.55	2013-2013	**69**	Maternal health	10	1.63	2015-2016
**22**	Cloud computing	22	1.73	2016-2016	**46**	Screening	13	1.19	2006-2011	**70**	Epidemiology	10	1.41	2001-2011
**23**	Body sensor networks	21	3.32	2007-2012	**47**	Quality of life	13	1.10	2010-2012	**71**	Intervention study	10	1.13	2013-2013
**24**	PDA[c]	20	8.65	2002-2012	**48**	Decision support system	13	1.08	2007-2008					

[a]TAM: technology acceptance model.

[b]ECG: electrocardiogram.

[c]PDA: personal digital assistants.
